# Internet Use and Knowledge of Clinical Trials Among US Adults: Cross-Sectional Survey Study

**DOI:** 10.2196/90967

**Published:** 2026-10-29

**Authors:** Zaib Hussain, Erin D Michos, Hailey N Miller, Cheryl R Himmelfarb, Payam Sheikhattari, Rifath Ara Alam Barsha, Timothy B Plante

**Affiliations:** 1 Division of Cardiology, Johns Hopkins School of Medicine, 600 N. Wolfe Street, Blalock 524C, Baltimore, MD, 21287, United States, 1 410-955-5999; 2 Department of Epidemiology, Johns Hopkins Bloomberg School of Public Health, Baltimore, MD, United States; 3 Welch Center for Prevention, Epidemiology, and Clinical Research, Johns Hopkins University, Baltimore, MD, United States; 4 Johns Hopkins School of Nursing, Baltimore, MD, United States; 5 Institute for Clinical and Translational Research, Johns Hopkins University, Baltimore, MD, United States; 6 Johns Hopkins Bloomberg School of Public Health, Baltimore, MD, United States; 7 Center for Urban Health Disparities Research and Innovation, Morgan State University, Baltimore, MD, United States; 8 School of Community Health and Policy, Morgan State University, Baltimore, MD, United States; 9 The Prevention Sciences Research Center, School of Community Health and Policy, Morgan State University, Baltimore, MD, United States; 10 Department of Medicine, Larner College of Medicine, University of Vermont, Burlington, VT, United States

**Keywords:** internet use, clinical trial knowledge, Health Information National Trends Survey 5 cycle 4, HINTS 5 cycle 4, health information, digital health, recruitment strategies, mobile internet, home-based internet, disparities, underrepresentation, health equity, clinical trials

## Abstract

**Background:**

Lack of knowledge about clinical trial (CT) research may perpetuate poor engagement of underrepresented populations. The rise of internet use may improve CT knowledge, and internet-based recruitment modalities might differentially benefit recruitment if internet users have higher baseline CT knowledge. We seek to bridge these gaps by assessing the relationship between internet use, access to health information, and CT participation.

**Objective:**

This study aimed to determine the association between internet use and CT knowledge among US adults.

**Methods:**

The Health Information National Trends Survey (HINTS) 5 cycle 4 is a cross-sectional, nationally representative survey of US adults. Participants self-reported internet use (yes or no) and knowledge of CTs (dichotomized as none or little vs higher). Logistic regression estimated odds ratios (ORs) for higher CT knowledge by internet use, adjusting for sociodemographic and health factors. Analysis was stratified by internet use modalities, exploring variations in knowledge.

**Results:**

Among a weighted sample of 249,896,898 participants (mean age 46, SD 17.0 years; 1471/2616, 50% women), 88% (n=2233) were internet users. No knowledge or only a little knowledge of CTs was reported among 97% (361/383) of internet nonusers and 89% (1919/2233) of internet users. There were significantly greater odds of higher CT knowledge among internet users than among nonusers in the fully adjusted model (OR 2.62, 95% CI 1.30-5.33; *P*=.009)**.** Stratified analyses demonstrated a positive association between combined mobile phone and home-based internet use and higher CT knowledge in the fully adjusted model (OR 2.69, 95% CI 1.34-5.37; *P*=.006).

**Conclusions:**

Internet use is associated with greater CT knowledge. Thus, internet-based recruitment modalities may augment conventional engagement methods to identify US adults potentially interested in CT participation.

## Introduction

Clinical trials (CTs) are critical for the advancement of evidence-based cardiovascular medicine. Despite their significance, participation in cardiovascular CTs is alarmingly low among many demographic groups, including women [1-4] and racial or ethnic minority groups [5,6]. This lack of representative participation results in a disconnect between the populations enrolled in these trials and the broader populations actually affected by cardiovascular diseases, limiting the generalizability of the outcomes [2]. Low CT engagement can be attributed to several factors, including a lack of awareness of research and information on available opportunities, mistrust of researchers and research institutions, and logistical barriers to participation [7].

Current research suggests that a substantial proportion of the population is unaware of ongoing CTs or lacks sufficient understanding of the purpose, process, or potential benefits of research [8,9]. This lack of comprehension may cause reluctance to enroll in research studies [8]. Knowledge of CTs is essential for optimizing participation.

The increasingly ubiquitous use of digital tools and platforms offers the opportunity to transform the dissemination of CT information, perceptions regarding CTs, and obtention of awareness among the general population and potentially augment recruitment of underrepresented groups [10-13]. The internet not only serves as a seemingly infinite repository for knowledge but also as a platform to disseminate information efficiently and broadly [14]. The utility of the internet has not been firmly established for the purposes of CT enrollment, and there is limited understanding of how to effectively harness it to improve participation in research. Preliminary data suggest that individuals who access health information through the internet have a better understanding of CTs and are more receptive to the research community [15]. However, there is a need for more comprehensive research within historically marginalized populations to elucidate the role of the internet, the modalities adults use to access CT information, and the associations between sociodemographic characteristics and the aforementioned factors [16].

Building on this gap, although prior studies have demonstrated an association between general internet use and health-related knowledge, associations between specific modalities of internet access and CT knowledge have not been well studied [17,18]. This distinction is increasingly important given the shift toward mobile-first internet access, particularly among populations historically underrepresented in CTs [19]. Moreover, although internet use is often assumed to improve health awareness, it remains unclear whether internet access is associated with greater CT knowledge at the population level [20]. Prior studies have also largely focused on general health literacy or awareness rather than CT knowledge specifically, which is a key determinant of research participation [21]. Therefore, our study extends prior work by examining whether differences in internet use and modality are associated with variation in CT knowledge.

Our study aims to bridge these knowledge gaps by quantifying the association between internet use and CT knowledge in a nationally representative sample. We additionally seek to examine the specific modalities adults use to access the internet and how these modalities relate to the acquisition of knowledge pertaining to CTs. This information may help inform the use of internet-based approaches to improve knowledge of research and willingness to participate in CTs among underrepresented populations.

## Methods

### Overview

The Health Information National Trends Survey (HINTS) 5 cycle 4, a cross-sectional, nationally representative survey, was conducted by the National Cancer Institute (NCI) among English- or Spanish-speaking, civilian, noninstitutionalized adults aged ≥18 years in the United States [22,23]. Cycle 4 of this self-administered, mail-based survey was conducted between February 24, 2020, and June 15, 2020. The survey included 3865 completed responses (response rate, 37%), and it was the first cycle to include questions on both CT knowledge and internet use. HINTS gathers data on the US population’s need for, access to, and use of health-related information, as well as their health-related behaviors, views, and knowledge [22].

The HINTS sampling design incorporates stratification based on the proportion of racial and ethnic minority populations within census tracts, as well as rural and urban distribution, to ensure adequate representation of diverse populations. Strata included high- and low-minority areas across both urban and rural settings, as defined by established census and rural-urban classification frameworks.

Oversampling of high-minority strata was performed by HINTS to improve the representation of underrepresented populations. Survey weights were applied to account for the complex sampling design and generate nationally representative estimates, incorporating demographic factors such as age, sex, educational attainment, marital status, race, ethnicity, and census region, as well as health-related factors including insurance and history of cancer. Additional details regarding the HINTS sampling methodology are available in the HINTS methodology report.

### Analytical Sample

We included respondents from HINTS 5, cycle 4 in our study. A total of 3977 questionnaires were available in the HINTS dataset. Of these, 112 (2.8%) were excluded due to general survey completion errors (40 [1%] were blank, 25 [0.6%] were incomplete, and 47 [1.2%] were duplicate surveys), and 93 (2.4%) participants were excluded due to errors in their responses to the CT knowledge question (92 [2.4%] did not answer the question, and 1 [<0.1%] provided an erroneous response). The final study sample consisted of 3772 respondents, representing a nationally weighted population of 249,896,898 individuals (Figure S1 in Multimedia Appendix 1).

### Exposure and Outcomes

Among those who reported internet use, we assessed CT knowledge as the primary outcome. It was evaluated using the following question from the survey: “Clinical trials are research studies that involve people. They are designed to compare new kinds of health care with the standard health care people currently get. For example, a new drug or a new way for patients to track their diets. How would you describe your level of knowledge about clinical trials?” Response options included “I don’t know anything about CTs,” “I know a little bit about clinical trials,” and “I know a lot about clinical trials.” For the analysis, this variable was dichotomized into a binary variable: participants without knowledge (“I don’t know anything” and “I know a little”) and those with knowledge (“*I know a lot*”).

Our primary exposure variable of interest was internet use, which was assessed in the survey through the following question: “Do you ever go online to access the Internet or World Wide Web, or to send and receive e-mail?” Participants could choose either “yes” or “no.” For the analyses, this variable was retained as a binary variable.

Our secondary exposure variable of interest was the modality used to access the internet, which was defined using the following questions. The first question asked, “How often do you access the internet through a computer at home?” and the second inquired, “How often do you access the internet on a mobile device (eg, cell phone, smartphone, or tablet)?”

We stratified the primary analysis by assessing CT knowledge among internet users according to their modality of internet access. Participants who answered “Daily” or “Sometimes” were considered internet users for that connection type (ie, home internet users or mobile device internet users), whereas those who answered “Never” or “Not applicable” were considered nonusers for that connection type. In the primary analysis, participants were categorized as internet users if they used either home or mobile internet. Participants who used neither home nor mobile internet were classified as internet nonusers. A secondary, mutually exclusive classification categorized respondents as neither home nor mobile internet users (“internet nonusers”), home internet users only, mobile device internet users only, or both home and mobile device internet users.

### Covariates

All covariates were self-reported. Age was categorized into 5 groups: 18 to 34 years, 35 to 49 years, 50 to 64 years, 65 to 74 years, and ≥75 years. Sex was recorded as either male or female, whereas sexual orientation was categorized as heterosexual, homosexual, bisexual, or something else. Race or ethnicity was categorized into groups labeled Hispanic, non-Hispanic White, non-Hispanic Black or African American, non-Hispanic Asian, and non-Hispanic multiple. BMI is the ratio of weight in kilograms to height in meters squared and was categorized as <18.5, 18.5 to <25, 25 to <30, 30 to <35, 35 to <40, or ≥40 kg/m^2^. Smoking status was categorized as current, former, or never. History of diabetes and history of hypertension were dichotomized as yes or no. Education was dichotomized as less than high school vs high school or greater to reflect a meaningful threshold in educational attainment associated with health literacy and access to health information and to ensure adequate sample sizes within categories for stable estimation [24,25]. Annual household income was categorized as ≤US $19,999, US $20,000 to US $34,999, US $35,000 to US $49,999, US $50,000 to US $74,999, and ≥US $75,000. Marital status was categorized as married, living with a partner, divorced, widowed, separated, or single. Employment status (derived from a survey item assessing occupational status) was dichotomized as employed vs not currently employed.

### Statistical Analysis

This analysis followed the approaches recommended by HINTS [22]. SE estimation used the jackknife resampling variance estimation method. It used a “delete-one” (JK1) approach, in which 1 of the 50 replicate deletion groups is dropped for each replicate and the estimates are recalculated using the remaining observations. These replicate estimates are then combined to produce the final variance estimate. Participants with missing data for any covariates were excluded from the analysis using a complete-case analytical approach.

Nonparametric bivariate demographic analyses were conducted leveraging demographic, socioeconomic, and health-related predictor variables to assess the relationship between these characteristics and internet use. The parametric testing included bivariate logistic regression to determine the association between internet use and the extent of CT knowledge among survey respondents. Initial unadjusted models estimated odds ratios (ORs) for higher CT knowledge among those who answered “yes” to internet use vs those who answered “no.” Subsequent models were adjusted sequentially for demographic, socioeconomic, and health-related factors to account for confounding.

Models were built progressively to assess changes in the association after adjustment for potential confounders and covariates. Model 1 was an unadjusted logistic regression model used to estimate the odds of higher CT knowledge among internet users vs internet nonusers. Model 2 was adjusted for key demographic factors, including age, sex, race or ethnicity, and sexual orientation. These variables were included to control for known demographic differences in internet use and CT knowledge that could confound the observed associations. Model 3 further adjusted for health-related factors, such as BMI, diabetes status, hypertension status, and smoking status. Model 4 was fully adjusted for socioeconomic factors that reflect the social determinants of health, including education, annual household income, marital status, employment status, and rural-urban residence.

Finally, stratification of the logistic regression was based on the modality of internet access used to access the World Wide Web, including mobile devices or home-based personal computers. This allowed a more sophisticated illustration of the association between the variables under observation. Stratified analyses were also adjusted for covariates in models 1 to 4.

A 2-tailed *P*<.05 was considered the threshold for statistical significance. All analyses were conducted using Stata (version 15; StataCorp LLC) [26].

### Ethical Considerations

This study did not require institutional review board (IRB) review because it involved secondary analysis of deidentified, publicly available data and was classified as “nonhuman subjects” research. Similarly, it did not require informed consent from survey respondents. The HINTS 5 cycle 4 general population survey was classified as nonhuman subjects research by the National Institutes of Health (NIH; NIH Office of Human Subjects Research; April 25, 2016 [Exempt #13204]) and underwent expedited IRB review (Westat IRB; March 28, 2016 [Project #6048.14]) [27].

## Results

Participant demographics and characteristics overall and by internet use are presented in Table 1.

Internet users comprised 88% (2233/2616) of the sample. Compared with internet nonusers, internet users were younger and more likely to be White, female, married, employed, and to have higher educational attainment and household income. They were also less likely to report diabetes, hypertension, or smoking. Despite having internet access, only about half of the participants reported some knowledge of CTs (Table 2).

**Table 1.** Demographic characteristics of the analytic population (weighted N=183,955,324)^a^.

Variables		Total participants (n=2616)	Internet nonusers (n=383)	Internet users (n=2233)	Internet users by access type		
					Home only (n=80)	Mobile only (n=125)	Both home and mobile (n=2023)
Weighted population estimate, n (%)		183,955,324 (100)	21,482,487 (12)	162,472,836 (88)	4,305,926 (2)	10,406,155 (6)	147,669,034 (80)
Age, years, n (%)							
	18‐34	416 (30)	13 (14)	403 (32)	1 (11)	25 (39)	377 (33)
	35‐49	549 (26)	35 (19)	514 (27)	2 (14)	34 (26)	478 (28)
	50‐64	811 (27)	99 (23)	712 (27)	13 (14)	41 (26)	656 (28)
	65‐74	579 (11)	114 (19)	465 (10)	36 (31)	23 (9)	404 (9)
	≥75	261 (6)	122 (26)	139 (3)	28 (30)	2 (0)	108 (3)
Race or ethnicity, n (%)							
	African American	320 (10)	66 (14)	254 (10)	5 (2)	13 (14)	235 (9)
	Asian	127 (5)	26 (9)	101 (4)	0 (0)	2 (2)	99 (5)
	Hispanic	428 (17)	102 (24)	326 (16)	2 (1)	29 (21)	295 (16)
	Multiple	90 (3)	9 (3)	81 (3)	0 (0)	6 (3)	75 (3)
	White	1651 (66)	180 (50)	1471 (68)	73 (97)	75 (61)	1319 (68)
Sex, n (%)							
	Male	1145 (51)	179 (58)	966 (50)	42 (68)	51 (52)	871 (50)
	Female	1471 (49)	204 (42)	1267 (50)	38 (32)	74 (48)	1152 (50)
Sexual orientation, n (%)							
	Heterosexual	2452 (93)	368 (95)	2084 (93)	78 (88)	119 (97)	1884 (93)
	Homosexual	64 (3)	3 (3)	61 (3)	0 (0)	2 (2)	59 (3)
	Bisexual	67 (3)	3 (1)	64 (3)	1 (1)	2 (0)	59 (4)
	Something else	33 (1)	9 (2)	24 (1)	1 (11)	2 (1)	21 (1)
BMI, kg/m², n (%)							
	<18.5	37 (1)	9 (2)	28 (1)	1 (1)	1 (0)	26 (1)
	18.5 to <25	817 (33)	114 (33)	703 (33)	27 (34)	34 (34)	640 (33)
	25 to <30	871 (32)	124 (38)	747 (31)	25 (27)	34 (19)	686 (32)
	30 to <35	514 (20)	68 (11)	446 (21)	22 (34)	35 (35)	388 (20)
	35 to <40	245 (9)	43 (10)	202 (9)	3 (4)	12 (6)	187 (9)
	≥40	132 (5)	25 (6)	107 (5)	2 (1)	9 (7)	96 (5)
History of diabetes, n (%)							
	Yes	506 (15)	130 (24)	376 (14)	21 (22)	15 (10)	339 (14)
	No	2110 (85)	253 (76)	1857 (86)	59 (78)	110 (90)	1684 (86)
History of hypertension, n (%)							
	Yes	1090 (34)	235 (49)	855 (33)	53 (52)	41 (22)	758 (33)
	No	1526 (66)	148 (51)	1378 (68)	27 (48)	84 (78)	1265 (67)
Smoking status, n (%)							
	Current	280 (13)	68 (19)	212 (12)	9 (8)	22 (25)	181 (12)
	Former	640 (23)	88 (20)	552 (23)	34 (51)	33 (20)	485 (22)
	Never	1696 (64)	227 (61)	1469 (65)	37 (41)	70 (56)	1357 (66)
Education, n (%)							
	Less than high school	146 (6)	89 (25)	57 (4)	1 (1)	13 (23)	43 (3)
	High school or greater	2470 (94)	294 (76)	2176 (96)	79 (99)	112 (77)	1980 (97)
Household income, n (%)							
	≤US $19,999	351 (12)	145 (35)	206 (9)	21 (29)	22 (13)	161 (8)
	US $20,000-34,999	306 (10)	80 (18)	226 (9)	20 (18)	15 (17)	190 (8)
	US $35,000-49,999	318 (12)	65 (19)	253 (11)	15 (16)	23 (11)	214 (11)
	US $50,000-74,999	473 (19)	50 (16)	423 (20)	7 (17)	21 (24)	394 (19)
	≥US $75,000	1168 (47)	43 (13)	1125 (52)	17 (21)	44 (36)	1064 (54)
Marital status, n (%)							
	Married	1333 (50)	142 (49)	1191 (50)	23 (27)	51 (34)	1117 (52)
	Living with partner	133 (4)	8 (1)	125 (5)	1 (1)	7 (5)	117 (5)
	Divorced	402 (8)	76 (7)	326 (8)	15 (10)	27 (11)	282 (8)
	Widowed	228 (4)	90 (12)	138 (3)	16 (11)	4 (2)	117 (2)
	Separated	57 (2)	11 (2)	46 (2)	1 (2)	6 (5)	39 (1)
	Single	463 (33)	56 (29)	407 (33)	24 (49)	30 (43)	351 (32)
Employment status, n (%)							
	Employed	1349 (58)	104 (36)	1245 (61)	17 (31)	76 (74)	1150 (61)
	Not currently employed	1267 (42)	279 (64)	988 (39)	63 (69)	49 (26)	873 (39)
Residence, n (%)							
	Urban	2322 (89)	323 (83)	1999 (90)	69 (86)	112 (95)	1814 (89)
	Rural	294 (12)	60 (17)	234 (11)	11 (14)	13 (5)	209 (11)

a Data are presented as unweighted counts (n) and survey-weighted percentages (%), unless otherwise indicated.

**Table 2.** Knowledge of clinical trials by internet use^a^.

Knowledge of clinical trials	Internet nonusers (n=383)	Internet users (n=2233)	Internet users by access type		
			Home internet only (n=80)	Mobile internet only (n=125)	Both home and mobile internet use (n=2023)
None, n (%)	235 (65)	619 (35)	26 (47)	48 (53)	544 (33)
A little, n (%)	126 (32)	1300 (54)	43 (45)	68 (39)	1186 (56)
A lot, n (%)	22 (3)	314 (11)	11 (8)	9 (8)	293 (12)

a Data are presented as unweighted counts (n) and survey-weighted percentages (%).

Participant characteristics also varied by internet modality (Table 1). Among internet users, the majority reported using both home and mobile internet. The age distribution was similar among individuals who used both modalities and those who used mobile internet only. Compared with those using only 1 modality of the internet, individuals who used both home and mobile internet were more likely to have higher household income and be married. Mobile-only users had a higher prevalence of chronic health conditions, including diabetes and hypertension, and were more likely to be employed. In comparison, respondents relying on home internet were more likely to identify as White and report higher levels of education.

Participants who used the internet were more likely to report having at least some knowledge of CTs than internet users (Table 2). Among internet users, individuals who used both home and mobile internet reported the highest levels of CT knowledge (1186/2023, 56% reported knowing a little; 293/2023, 12% reported knowing a lot). These proportions were smaller among those who used home internet only (43/80, 45% reported knowing a little; 11/80, 8% reported knowing a lot) and mobile internet only (68/125, 39% reported knowing a little; 9/125, 8% reported knowing a lot). CT knowledge was lowest among those who did not use the internet (235/383, 65% reported no knowledge; 126/383, 32% reported knowing a little; and 22/383, 3% reported knowing a lot).

As shown in Figure 1 and Table S1 (Multimedia Appendix 1), the unadjusted regression model (model 1) demonstrated that participants who used the internet had significantly higher odds of higher CT knowledge (OR 3.63, 95% CI 2.11-6.27; *P*<.001). The association between internet use and CT knowledge remained significant in further models that accounted for demographic and health-related factors. Model 2 (OR 3.14, 95% CI 1.67-5.91; *P*=.001) was adjusted for demographic factors, including age, race or ethnicity, sex, and sexual orientation, whereas model 3 (OR 3.59, 95% CI 1.81-7.15; *P<*.001) accounted for health-related variables, including BMI, history of diabetes, history of hypertension, and smoking status. The association was attenuated but remained statistically significant in the fully adjusted model 4 (OR 2.62, 95% CI 1.30-5.33; *P*=.oo9).

We also examined the association between the modality of internet use and higher CT knowledge (Table 3).

**Figure 1.**
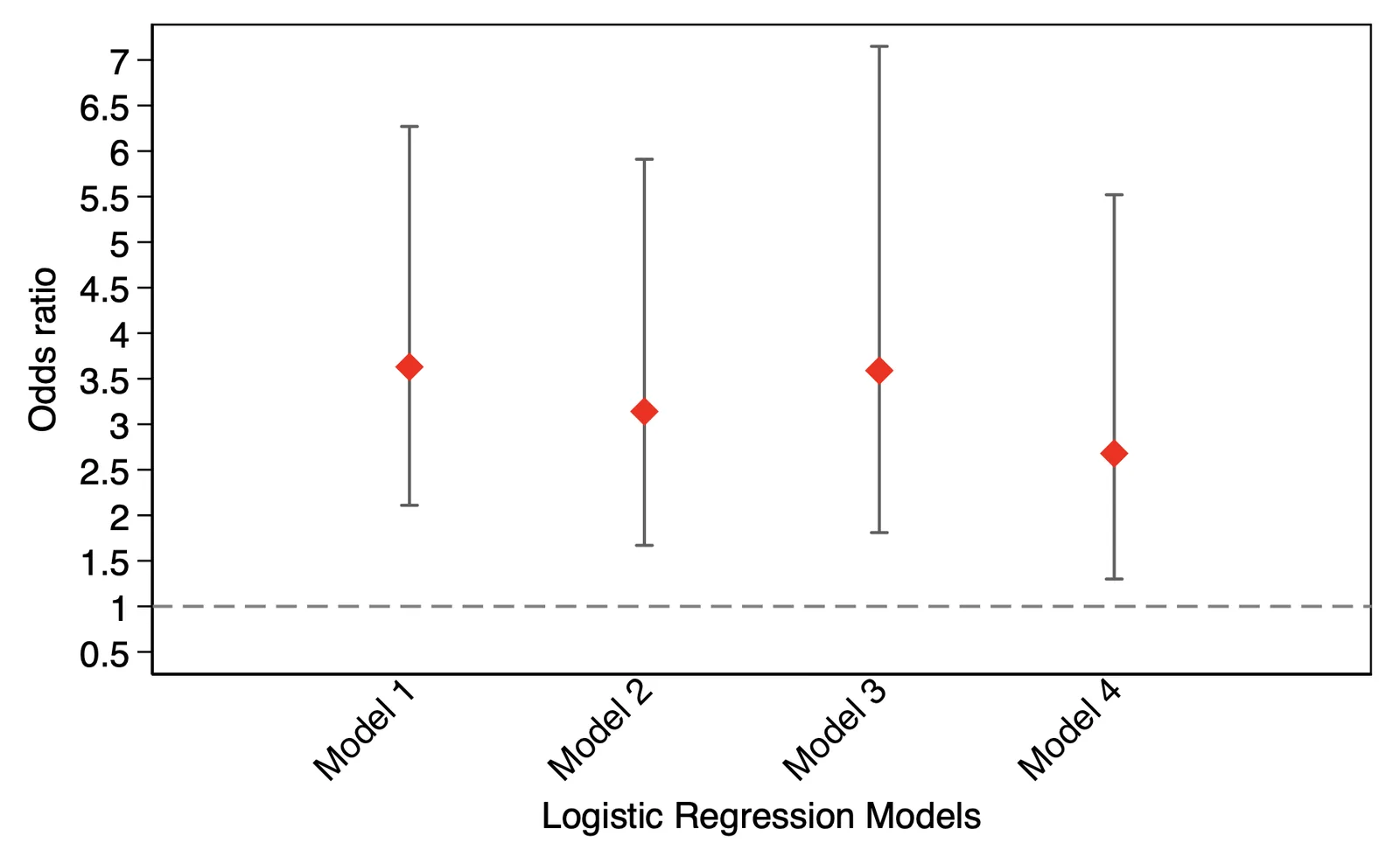
Odds ratios for higher clinical trial knowledge among internet users compared with internet nonusers (reference). Model 1 was unadjusted. Model 2 was adjusted for age, sex, race or ethnicity, and sexual orientation. Model 3 was adjusted for the variables in model 2 plus BMI, diabetes status, hypertension status, and smoking status. Model 4 was adjusted for the variables in model 3 plus education, household income, marital status, employment status, and rural-urban residence. Error bars indicate 95% CIs; the dashed horizontal line indicates an OR of 1.

**Table 3.** Odds ratios (ORs) for having higher clinical trial knowledge by internet use modality.

Models	Internet nonusers	Home internet use only, OR (95% CI)	Mobile internet use only, OR (95% CI)	Both home and mobile internet use, OR (95% CI)
Model 1^a^	Reference	2.52 (0.61-10.44)	2.49 (0.42-14.85)	3.75 (2.19-6.42)
Model 2^b^	Reference	2.40 (0.54-10.61)	2.33 (0.35-15.26)	3.23 (1.73-6.03)
Model 3^c^	Reference	2.70 (0.61-11.99)	2.75 (0.38-19.80)	3.68 (1.87-7.26)
Model 4^d^	Reference	2.14 (0.45-10.24)	2.38 (0.34-16.65)	2.69 (1.34-5.37)

a Model 1: unadjusted.

b Model 2: adjusted for age, sex, race or ethnicity, and sexual orientation.

c Model 3: model 2 plus BMI, diabetes status, hypertension status, and smoking status.

d Model 4: model 3 plus education, household income, marital status, employment status, and rural-urban residence.

Both mobile phone and home-based internet use were positively correlated with higher CT knowledge. The likelihood of higher CT knowledge was greater among individuals who used both mobile phone and home-based internet than among internet nonusers in the fully adjusted model 4 (OR 2.69, 95% CI 1.34-5.37). This OR was similar to those observed for home internet use only vs internet nonusers and mobile internet use only vs internet nonusers, although the CIs were wide and included the null.

## Discussion

### Key Findings

In this nationally representative sample of US adults, internet users had greater knowledge of CTs than internet nonusers. This finding aligns with other research that has demonstrated a positive correlation between internet use and greater health literacy, encompassing awareness of CTs [10,28]. Our findings indicate that users of both mobile and home internet had approximately 2.7 times the odds of having higher CT knowledge, underscoring the relevance of varied online platforms for disseminating health information [29]. Notably, individuals engaging with both home and mobile internet demonstrated the highest likelihood of reporting CT knowledge, suggesting that multimodal digital engagement may reflect greater exposure to health information. These findings suggest that internet access modality may be relevant when developing digital health strategies to engage consumers. In particular, CT knowledge interventions targeting users of both home and mobile internet might yield better results, as these individuals may be more receptive to and accessible through digital outreach efforts aimed at improving CT awareness. The results of this study expand on the existing body of research showing the importance of harnessing the internet as a means of improving general understanding of health issues in the community.

Previous scientific literature has shown that using the internet is linked to improved knowledge of health. People who use mobile internet to access health information have greater overall health literacy [30,31]. However, few studies have investigated specific methods of internet access, and their focus has not been specifically on CT knowledge [9,10]. In comparison, our study highlights that both mobile and home internet access are correlated with higher CT knowledge. This is especially pertinent considering the growing dependence on mobile devices for internet access among underrepresented populations in CTs, including low-income individuals and racial or ethnic minority groups, as reported by the Pew Research Center [32]. These populations are more likely to rely on smartphones for internet access, indicating that mobile-optimized digital strategies could be promising for improving knowledge of CTs [33].

Another implication of our findings concerns the persistent underrepresentation of women and racial or ethnic minority groups in CTs. Research shows that these groups are often represented at lower rates in CTs, thereby constraining the generalizability of trial outcomes to the wider community impacted by cardiovascular disorders [3]. Accessible online platforms may offer opportunities to enhance CT knowledge among populations with historically lower participation, and digital outreach may contribute to narrowing long-standing disparities. This, in turn, may improve the generalizability of trial outcomes [34]. However, although necessary, knowledge alone may be insufficient to drive participation in CTs. Further research is needed to determine whether improvements in CT knowledge through digital means translate into higher enrollment and more representative participation in CTs.

Our findings lay a solid foundation for future research focused on elucidating the connection between internet use and participation in CTs. Longitudinal studies capable of mapping changes in CT knowledge over time and establishing causal relationships between internet use and knowledge would be beneficial. Moreover, prospective research should explore how the content and quality of online material influence public knowledge of CTs. The importance of providing precise and accessible digital health content, especially for groups with lower digital literacy, cannot be overemphasized. Subsequently, studies should focus on defining valid and optimal methods for disseminating CT information via digital platforms [35]. This may include customizing content to meet the needs of various audiences. Further extrapolation can benefit the efficacy of digital health treatments, thereby enhancing CT understanding and mitigating other obstacles to CT involvement.

The application of a sizable, weighted sample typical of the general US population is one of the main strengths of this study. This improves the generalizability of our results to the broader US population. Furthermore, the emphasis of the study on particular internet modalities rather than overall internet use offers a more complex understanding of how various types of digital participation are associated with health knowledge. Thorough control for possible confounders further strengthens the validity of the established association. Our findings, however, need to be considered in the context of several study limitations. The cross-sectional observational design prevents the determination of causation; therefore, we cannot say with certainty that internet use generates better CT knowledge. Furthermore, recall bias or social desirability bias in self-reported measures of internet use and CT knowledge could affect the accuracy of the results. Moreover, the study did not assess the type or quality of information obtained online, which can differ greatly among consumers. Residual confounding could also explain the observed associations. The data were collected in 2020 and may not fully reflect current patterns of internet use, particularly given rapid changes in digital access and behavior following the COVID-19 pandemic. However, HINTS 5 cycle 4 was selected because it is the most recent cycle that includes both detailed measures of CT knowledge and modality-specific internet use variables. Subsequent HINTS cycles did not include these measures in a comparable manner. Specifically, HINTS 6 did not assess CTs, and although HINTS 7 included questions related to CTs, it did not assess CT knowledge, which was the primary outcome of this study. Therefore, HINTS 5 cycle 4 represents the most appropriate dataset to address our research question. Importantly, the observed relationships between internet use, modality of access, and CT knowledge are likely to remain relevant, as disparities in digital access and health information engagement persist over time.

Our study provides a foundation for future studies exploring the association between internet use and CT enrollment. Further studies using longitudinal approaches that can more effectively establish causal links between internet use and CT knowledge might be beneficial. Research should also examine how certain online tools and the quality of online materials shape public knowledge of CTs. Moreover, considering the growing use of mobile devices, additional research is required to determine how best to maximize mobile health solutions to improve CT knowledge and involvement [34]. Ensuring equitable access to health information also depends on the inclusion of people with low digital literacy [36].

### Conclusions

In sum, our study emphasizes the positive association between internet use and CT knowledge, especially with regard to mobile phone and home-based internet access. After accounting for demographic and health-related covariates, general internet use was associated with higher CT knowledge. Furthermore, both home and mobile modalities of internet access were identified to have a positive correlation with higher CT knowledge. These results suggest that individuals with consistent internet access who engage through multiple modalities may be more informed about research and potentially more amenable to recruitment efforts. These findings support further evaluation of focused digital interventions aimed at increasing the engagement of trial-ready populations in clinical research, thereby helping the advancement of medical science. Subsequent ventures must concentrate on exploring means to effectively reach groups with limited digital engagement to promote inclusion and equity in CT participation. A visual overview of the study design and key findings is provided in Multimedia Appendix 2.

## Supplementary material

10.2196/90967 Multimedia Appendix 1 Odds ratios for higher clinical trial knowledge across progressively adjusted models and participant inclusion flow diagram.

10.2196/90967 Multimedia Appendix 2 Visual abstract

## Abbreviations

CT: clinical trial
HINTS: Health Information National Trends Survey
IRB: institutional review board
NCI: National Cancer Institute
NIH: National Institutes of Health
OR: odds ratio
