# Supplementary material for: Internet Use and Knowledge of Clinical Trials Among US Adults: Cross-Sectional Survey Study
Source: Online J Public Health Inform. 2026 Oct 29;18:e90967. doi: 10.2196/90967 (PMC13718384; doi:10.2196/90967)
Supplement: Supplementary file 1 [file ojphi-v18-e90967-s001.docx]

**Multimedia Appendix 1: Supplementary Materials**

**Supplementary Table 1: Odds Ratios for high clinical trial knowledge from progressively adjusted models***

|  | **OR (95%CI)** | | | |
| --- | --- | --- | --- | --- |
| **Covariates** | **Model A** | **Model B** | **Model C** | **Model D** |
| **Internet Use** | 3.63 (2.11-6.27) | 3.14 (1.67-5.91) | 3.59 (1.81-7.15) | 2.62 (1.30 – 5.33) |
| **Age, y** |  | 0.99 (0.98 – 1.00) | 1.00 (0.99 – 1.01) | 1.00 (0.99 – 1.01) |
| **Race/Ethnicity** |  |  |  |  |
| White |  | Ref | Ref | Ref |
| Hispanic |  | 0.65 (0.28 – 1.53) | 0.61 (0.23 – 1.59) | 0.66 (0.25 – 1.76) |
| African American |  | 0.67 (0.34 – 1.31) | 0.72 (0.34 – 1.51) | 0.71 (0.31 – 1.60) |
| Asian |  | 1.73 (0.77 – 3.89) | 1.44 (0.64 – 3.28) | 1.37 (0.61 – 3.11) |
| Other |  | 0.42 (0.23 – 1.29) | 0.48 (0.22 – 1.06) | 0.55 (0.24 – 1.24) |
| **Sex** |  |  |  |  |
| Male |  | Ref | Ref | Ref |
| Female |  | 0.93 (0.72 – 1.28) | 0.85 (0.68 – 1.17) | 0.94 (0.67 - 1.33) |
| **Sexual Orientation** |  |  |  |  |
| Heterosexual |  | Ref | Ref | Ref |
| Homosexual |  | 1.35 (0.47 – 3.83) | 1.53 (0.47 – 4.56) | 1.40 (0.42 – 4.62) |
| Bisexual |  | 2.98 (1.23 – 7.27) | 2.77 (1.05 – 7.48) | 2.85 (0.93 – 8.73) |
| Something Else |  | 0.83 (0.26 – 3.10) | 0.84 (0.23 – 3.29) | 0.85 (0.17 – 4.40) |
| **BMI, kg/m^2^** |  |  |  |  |
| <18.5 |  |  | Ref | Ref |
| 18.5 to <25 |  |  | 0.21 (0.05 – 0.90) | 0.20 (0.04 – 0.96) |
| 25 to <30 |  |  | 0.18 (0.04 – 0.78) | 0.16 (0.03 – 0.78) |
| 30 to <35 |  |  | 0.15 (0.03 – 0.74) | 0.14 (0.02 – 0.78) |
| 35 to <40 |  |  | 0.17 (0.03 – 0.91) | 0.15 (0.03 – 0.94) |
| ≥40 |  |  | 0.09 (0.02 – 0.47) | 0.08 (0.01 – 0.50) |
| **History of Diabetes** |  |  |  |  |
| Yes |  |  | Ref | Ref |
| No |  |  | 1.02 (0.37 – 2.83) | 1.08 (0.39 – 3.00) |
| **History of Hypertension** |  |  |  |  |
| Yes |  |  | Ref | Ref |
| No |  |  | 0.86 (0.52 – 1.43) | 1.00 (0.56 – 1.48) |
| **Smoking Status** |  |  |  |  |
| Current |  |  | Ref | Ref |
| Former |  |  | 0.84 (0.24 – 2.95) | 0.76 (0.23 – 2.58) |
| Never |  |  | 1.19 (0.36 – 3.97) | 1.07 (0.32 – 3.53) |
| **Education** |  |  |  |  |
| Less than high school |  |  |  | Ref |
| High School or greater |  |  |  | 3.01 (1.09 – 8.33) |
| **Household Income** |  |  |  |  |
| ≤$19,999 |  |  |  | Ref |
| $20,000 to $34,999 |  |  |  | 0.81 (0.35 – 1.89) |
| $35,000 to $49,999 |  |  |  | 0.68 (0.26 – 1.80) |
| $50,000 to $74,999 |  |  |  | 0.64 (0.31 – 1.30) |
| ≥$75,000 |  |  |  | 1.02 (0.47 – 2.20) |
| **Marital Status** |  |  |  |  |
| Married |  |  |  | Ref |
| Living with partner |  |  |  | 1.63 (0.54 – 4.94) |
| Divorced |  |  |  | 1.00 (0.52 – 1.89) |
| Widowed |  |  |  | 0.74 (0.32 – 1.71) |
| Separated |  |  |  | 0.76 (0.10 – 6.14) |
| Single (never married) |  |  |  | 1.02 (0.62 – 2.12) |
| **Employment Status** |  |  |  |  |
| Employed |  |  |  | Ref |
| Not currently employed |  |  |  | 1.55 (0.93 – 2.59) |
| **Residence** |  |  |  |  |
| Urban |  |  |  | Ref |
| Rural |  |  |  | 0.71 (0.33 – 1.52) |

*Model A: Unadjusted. Model B: Age, sex, race/ethnicity, and sexual orientation. Model C: Model B plus BMI, diabetes status, hypertension status, and smoking status. Model D: Model C plus education, household income, marital status, employment status, and rural-urban residence.

**Supplementary Figure 1: Inclusion flow diagram**
